# The effects of carotid plaque classification and bifurcation angle on plaque: a computational fluid dynamics simulation

**DOI:** 10.3389/fphys.2025.1509875

**Published:** 2025-03-21

**Authors:** Ai Chen, Zhuo Chen, Jun Su, Jie Pen, Tao Luo, Hua Zhong

**Affiliations:** ^1^ Department of Neurosurgery, Nanchuan Hospital, Chongqing Medical University, Chongqing, China; ^2^ Department of Pain Management, Mianyang 404 Hospital, Mianyang, Sichuan, China

**Keywords:** carotid artery bifurcation, hemodynamics, plaque formation, shear stress, vascular bifurcation angle

## Abstract

**Objectives:**

To investigate the influence of plaque distribution and vascular bifurcation angle on hemodynamics within the carotid artery bifurcation and to explore the role these factors play in the development of vulnerable carotid plaques. The study aims to provide a more comprehensive understanding of how complex hemodynamic patterns affect plaque formation, vulnerability, and progression.

**Methods:**

Patient-specific carotid bifurcation models were reconstructed using 3D rotational angiography and CT angiography, validated by digital subtraction angiography. Computational fluid dynamics (ANSYS Fluent) with non-Newtonian modeling simulated hemodynamics under patient-specific boundary conditions. Plaque morphology and hemodynamic parameters (TAWSS, OSI, ECAP) were quantified. Statistical analyses included Spearman’s correlations and non-parametric tests for bifurcation angles/plaque locations.

**Results:**

Numerical simulations demonstrated that plaque subtypes and bifurcation angles critically modulate carotid hemodynamics. Elevated wall shear stress (WSS) upstream of plaques (sites M/N) increased rupture susceptibility, whereas low WSS at the outer bifurcation (site P) exacerbated atherogenesis. Larger bifurcation angles reduced peak velocities, expanded low-velocity zones, and diminished WSS, amplifying atherosclerosis risk. Vortex-driven low-shear regions prolonged platelet residence, enhancing thrombotic propensity. Fluid-structure interactions revealed arterial wall deformation near bifurcations, correlating with endothelial injury and plaque progression. These hemodynamic alterations underscore the biomechanical interplay driving plaque vulnerability and thrombosis in carotid atherosclerosis.

**Conclusion:**

Carotid plaque vulnerability arises from bifurcation angle-dependent hemodynamic disturbances, where elevated upstream wall shear stress predisposes to rupture, while low-shear zones at the outer bifurcation accelerate atherogenesis. Vortex-driven platelet retention and fluid-structure interactions exacerbate endothelial dysfunction, underscoring hemodynamic targeting for clinical risk mitigation.

## 1 Introduction

The carotid arterial system, comprising the common carotid artery (CCA), internal carotid artery (ICA), and external carotid artery (ECA), is vital for perfusing the brain, head, and neck (Sethi et al., 2023). At the carotid bifurcation, atherosclerotic plaques frequently form, posing significant risk for cerebrovascular events (AbuRahma, 2021). Plaques are classified based on morphology (fibrous, lipid-rich, calcified, ulcerated), hemodynamics (mild <50%, moderate 50%–69%, severe ≥70% stenosis), and rupture risk (stable, vulnerable, thrombogenic) (Mura et al., 2020). Carotid atherosclerosis ranks among the most prevalent cardiovascular and cerebrovascular diseases, primarily leading to stroke (Tang et al., 2022). Atherosclerosis and plaques primarily affect large and medium-sized vessels with complex geometric shapes, frequently identify at the carotid bifurcation (ansen et al., 2020). Plaques can obstruct distal vascular blood supply through growth and rupture. Branching causes axial flow to follow a curved path, resulting in the formation and separation of secondary flows. The ICA sinus at the far end of the branch point is prone to plaque formation. Studies have confirmed that regions with low and highly oscillatory local wall shear stress (WSS) in vessels are more susceptible to atherosclerosis and plaque development, while high WSS in narrow areas can cause plaque rupture and thrombosis (Zhou et al., 2023). Wall shear stress distribution within the carotid bifurcation is spatiotemporally dynamic and complex, with local hemodynamic properties playing a pivotal role in atherosclerosis formation and progression. Therefore, studying the influence of hemodynamic characteristics on atherosclerosis formation and vulnerability development is essential. In addition, computational fluid dynamics (CFD) can be used to study the fluid and heat transfer in physical models, and it is an effective calculation method to analyze the fluid flow in pipes (Ren et al., 2025a).

Factors such as the degree of vascular stenosis, branch structure, and plaque classification can significantly affect blood flow characteristics. The degree of luminal stenosis is classified as mild (less than 50%), moderate (50%–69%), and severe (70% or more) (Ahmadpour et al., 2021)^.^ When significant stenosis (over 75%) occurs, carotid endarterectomy (CEA) is required (Kiwan et al., 2022). Gao et al. (2009) found that 8.16% of unstable plaques were found in mild and moderate stenosis, while 2.1% were found in severe stenosis, indicating that unstable plaques are more common in moderate stenosis than in severe stenosis, and 54% of plaques in moderate stenosis are unstable plaques. Plaques commonly occur in regions with complex flow patterns near the bifurcation ridge and on the opposite side, often exhibiting instability. Previous studies have highlighted shear stress as pivotal in plaque initiation and progression (Hartman et al., 2021). Excessive shear stress reduces platelet-derived growth factor secretion, inhibits protein synthesis in vascular smooth muscle cells, promotes apoptosis, and increases plaque vulnerability. Conversely, low shear stress is a primary factor in atherosclerotic plaque formation. Low shear stress can reduce the secretion of endothelial protective factors, contain more macrophages, lipids, metalloproteinases (MMPs), and reduce the number of vascular smooth muscle cells (VSMC), promote the secretion of vascular injury factors, and imbalance the secretion of NO, endothelin, prostacyclin II, angiotensin II and other bioactive molecules, leading to vascular endothelial damage, inducing vascular endothelial dysfunction, and promoting the formation of atherosclerosis (He et al., 2022).

Moreover, with significant advancements in computer science over recent decades, computer simulation technology has emerged as a powerful tool in engineering and academic research (Ren et al., 2025b; Hu et al., 2019; Oliveira et al., 2019; Ren et al., 2024). In the study of atherosclerotic plaque formation, researchers have conducted numerous hemodynamic numerical simulations. Bourantas et al. (2020) found that the distribution of endothelial shear stress (ESS) is an independent factor driving the progression of carotid atherosclerosis through computational fluid dynamics (CFD) modeling and multimodal imaging. Local hemodynamics and plaque formation exhibit nonlinear changes during atherosclerosis progression, with the rate of plaque thickness increase varying across different hemodynamic regions (Li et al., 2022). Bennati et al. (2021) carried out a three-dimensional (3D) fluid-structure interaction (FSI) simulation of atherosclerosis, and compared some fluid dynamics and structural quantities of 15 patients with different types of plaque characteristics. The results show that the morphology and mechanical properties of different patch components play an important role in determining the vulnerability of patches. Domanin et al. (2019) explored the possibility of hemodynamic disorder and geometric features to predict long-term carotid restenosis after carotid endarterectomy (CEA). Studies have shown that the wide widening of the carotid ball should be avoided in the repair of arteriotomy, which is related to restenosis by producing flow obstacles. Hemodynamics and geometry-based analysis provide the possibility for preoperative planning, guiding clinical decision-making and risk stratification of late restenosis. However, further investigation into plaque vulnerability is still necessary.

Therefore, this paper employs computational fluid dynamics (CFD) methods to investigate the flow field, pressure field, and shear stress characteristics of pulsatile blood flow in bifurcation vessels, predicting pressure, shear stress, and inner wall deformation at key locations. From a hemodynamic perspective, the influence of two critical factors, plaque type and vascular bifurcation, on the vulnerability of atherosclerotic plaques is analyzed.

## 2 Simulation details

### 2.1 Geometric modeling

Three-dimensional rotational angiography (3D-RA) was performed using a Siemens Artis dBA Twin system, complemented by high-resolution computed tomography angiography (CTA) (Tsuji et al., 2020). Digital subtraction angiography (DSA) served as the gold standard to validate anatomic fidelity, ensuring <1 mm spatial resolution and <5% geometric distortion through landmark-based registration. Segmentations of the carotid bifurcation (common carotid artery [CCA], carotid bulb [CB], internal carotid artery [ICA], and external carotid artery [ECA]) were reconstructed in Geomagic Wrap (v2021, 3D Systems) using automated thresholding followed by manual refinement by two independent raters (κ = 0.92, p < 0.001). Plaque characteristics—morphology (ulceration, surface irregularity), volume, calcification (Agatston score >100), and lipid-rich necrotic core (LRNC) extent—were quantified using validated CTA protocols (Gholipour et al., 2018).

### 2.2 Computational fluid dynamics (CFD) modeling

#### 2.2.1 Imaging acquisition and 3D model reconstruction

Imaging Protocol: High-resolution 3D rotational angiography (CTA) and digital subtraction angiography (DSA) were performed using an Artis dBA Twin system (Siemens Healthineers). DSA images served as the gold standard for validating anatomical accuracy and spatial resolution of CTA-derived data. To enhance reproducibility, scan parameters were standardized (tube voltage: 80 kV, contrast medium: Iohexol [350 mgI/mL], injection rate: 4 mL/s).

Segmentation and Meshing: Anatomical segmentation of the carotid bifurcation region, including plaques, was performed using Geomagic Wrap (3D Systems) with adaptive thresholding to account for calcification density gradients. Plaque morphology (length, thickness) and bifurcation angles were quantified using semi-automated algorithms. To mitigate user-dependent bias, three independent operators validated segmentation consistency (interclass correlation coefficient >0.85). The reconstructed 3D model was meshed in ANSYS Fluent with hybrid polyhedral elements (3.5 million cells), refined near bifurcations and plaque surfaces to resolve boundary layers (y+ < 1). Mesh independence was confirmed via grid convergence index (GCI <5%).

#### 2.2.2 Hemodynamic simulations

Boundary Conditions: Patient-specific velocity waveforms from Doppler ultrasound were prescribed at the common carotid artery (CCA) inlet. Outlet pressure boundary conditions for the internal (ICA) and external (ECA) carotid arteries were derived from brachial artery pressure measurements. To address inter-patient variability, parametric inlet velocities (0.3–0.7 m/s) were tested under pulsatile flow (Womersley number: 4.2).

Fluid Dynamics: Blood was modeled as a non-Newtonian fluid using the Carreau-Yasuda model (μ∞ = 3.45 mPa s, μ0 = 56 mPa s, λ = 3.313 s) to better capture shear-thinning behavior. Laminar flow assumptions were validated by calculating Reynolds numbers (<1,200 in all branches). Discrete phase modeling (DPM) was integrated to track platelet trajectories (10,000 particles, diameter = 3 μm). Particle residence time (PRT) and near-wall stagnation zones were quantified using Lagrange particle tracking.

Validation: Digital Subtraction Angiography (DSA) is used to validate blood flow distribution patterns, particle tracking data, and hemodynamic parameters, with the aim of improving the accuracy of clinical predictions in hemodynamic models.

#### 2.2.3 Plaque risk stratification

Hemodynamic parameters—time-averaged wall shear stress (TAWSS), oscillatory shear index (OSI), and endothelial cell activation potential (ECAP)—were calculated at plaque surfaces. Risk classification was defined as:

High risk: TAWSS <0.5 Pa, OSI >0.3, ECAP >1.5.

Medium risk: TAWSS = 0.5–1.5 Pa, OSI = 0.1–0.3.

Low risk: TAWSS >1.5 Pa, OSI <0.1.

Plaque vulnerability was further correlated with geometric features (ulceration depth/width ratio, cap thickness) using multivariate logistic regression.

**FIGURE 1:**
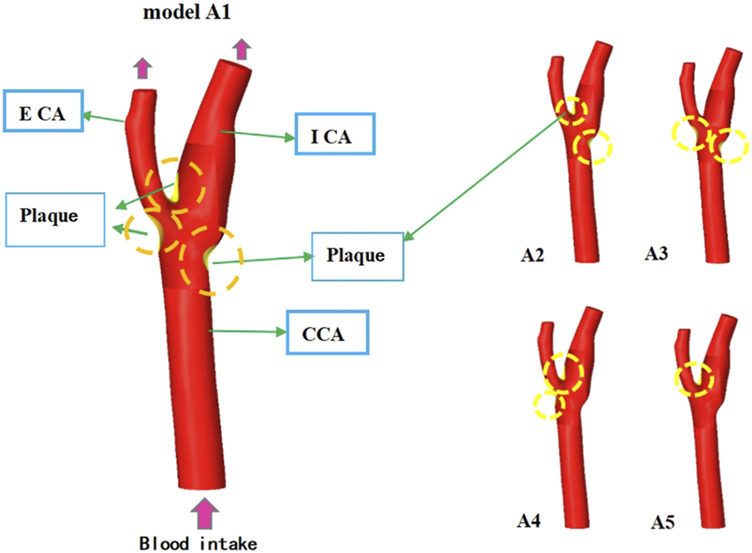
Model of plaques at different locations of the strong artery.

As shown in Figure 1, the schematic diagram of different plaque classification models shows that Model A1 is located at the bifurcation ridge, and there are plaque areas on the outer side of Internal carotid artery (ICA) and External carotid artery (ECA). Model A2 plaques are found at the bifurcation ridge and on the outer side ICA. In Model A3, features plaques located on the outer sides of ICA and ECA, while in Model A4, plaques are located on the outer side of ECA and at the bifurcation ridge. Model A5 exhibits plaques solely at the bifurcation ridge.

#### 2.2.4 Parametric bifurcation angle analysis

**FIGURE 2:**
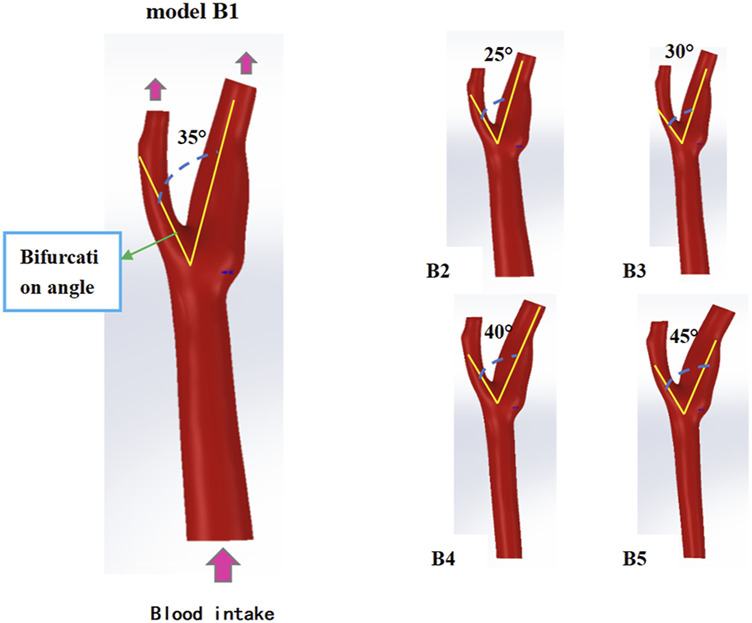
Model of different bifurcation angles of carotid artery vessels.

Five bifurcation angles (25°–45°) Figure 2 were modeled by incrementally adjusting ICA-ECA angulation in SolidWorks (Shen et al., 2021). Computational domains retained identical CCA diameter (6.35 mm) (Table 1) to isolate flow-angle relationships. Vortex formation time (VFT) and Dean number were computed to assess flow instability and secondary flow intensity.

**TABLE 1:** Vascular model geometric parameters (mm).

Parameter	Common carotid artery (CCA)	Internal carotid artery (ICA)	External carotid artery (ECA)
Vascular diameter	6.35	4.5 (extremity)	3 (extremity)
Blood vessel length	32	23.09	19.35
Plaque length	7	5.12	4
Average thickness of plaques	1	2	2

### 2.3 Mathematical model

A three-dimensional unsteady flow model is established, considering blood as a viscous, uniform, and incompressible Newtonian fluid, with blood flow assumed to be laminar. The model for blood flow, based on the Navier-Stokes equation and continuity equation, is as follows (Ren F et al., 2025).

$$\left\{\begin{array}{l} \rho \left[\frac{\partial U}{\partial t}+\left(U\cdot \nabla \right)U\right]=\nabla \cdot \left[-pI+\left({\nabla U+\left(\nabla U\right)}^{T}\right)\right]\\ \nabla \cdot U=0 \end{array}\right\}$$

In the formula: U is the blood velocity; density ρ = 1.06 × 10^3^ kg/m^3^; Dynamic viscosity coefficient μ = 3–4 mPa s。

The pressure at the vascular outlet is set to 13,332 Pa, and a velocity boundary condition is applied at the vascular inlet. To ensure that the simulation results closely mimic real physiological conditions, the inlet velocity (unit: m/s) varies with the cardiac pulsation cycle. The expression for this variation is as follows:

$$u_{m}\left(t\right)=\left\{\begin{array}{l} 0.5\times \sin\left(4\times \pi \times \left(t+0.0160236\right)\right),0\leq t\leq 0.218\\ 0.1,0.218<t\leq 1 \end{array}\right\}$$

### 2.4 Parameter settings

ANSYS software was utilized for meshing and solving equations. The geometric model was meshed using tetrahedral elements, with the boundary layer divided into five layers to ensure the reliability of the study results. To guarantee the accuracy of the numerical simulation, mesh independence verification was performed based on shear stress at the bifurcation point. The mesh sizes for models A1 to A5 were 614,218, 564,799, 492,445, 493,648, and 455,421, respectively, while the mesh sizes for models B1 to B5 were 380,878, 458,504, 359,058, 404,982, and 489,691, respectively. The simulation error when further increasing the mesh size was within 3%, confirming that the selected mesh sizes were reasonable for this study.

### 2.5 Statistical analysis method

Parametric and non-parametric statistical methods were used to assess the effects of bifurcation angle and plaque location on key haemodynamic parameters, including velocity, wall shear stress (WSS) and pressure. For the analysis of the continuous variable bifurcation angle, Spearman’s correlation analysis was used to explore the correlation between bifurcation angle (X-axis) and haemodynamic parameters (Y-axis), and the results are reported within 95% confidence intervals. Plaque location was used as a discontinuous variable, and non-parametric tests were used to assess the effect of different locations on the distribution of haemodynamic parameters. As the data showed a non-normal distribution, appropriate non-parametric statistics were chosen to analyse the differences in velocity, WSS and pressure in predefined regions of interest, including the upstream region, bifurcation ridge and lateral fossa. Descriptive statistics were used to summarise the mean and standard deviation of haemodynamic parameters in each region. In addition, multivariate simulation analysis was performed to explore the correlation between plaque morphology and local haemodynamic pressures.

## 3 Results

### 3.1 The impact of different plaque subtypes

**FIGURE 3:**
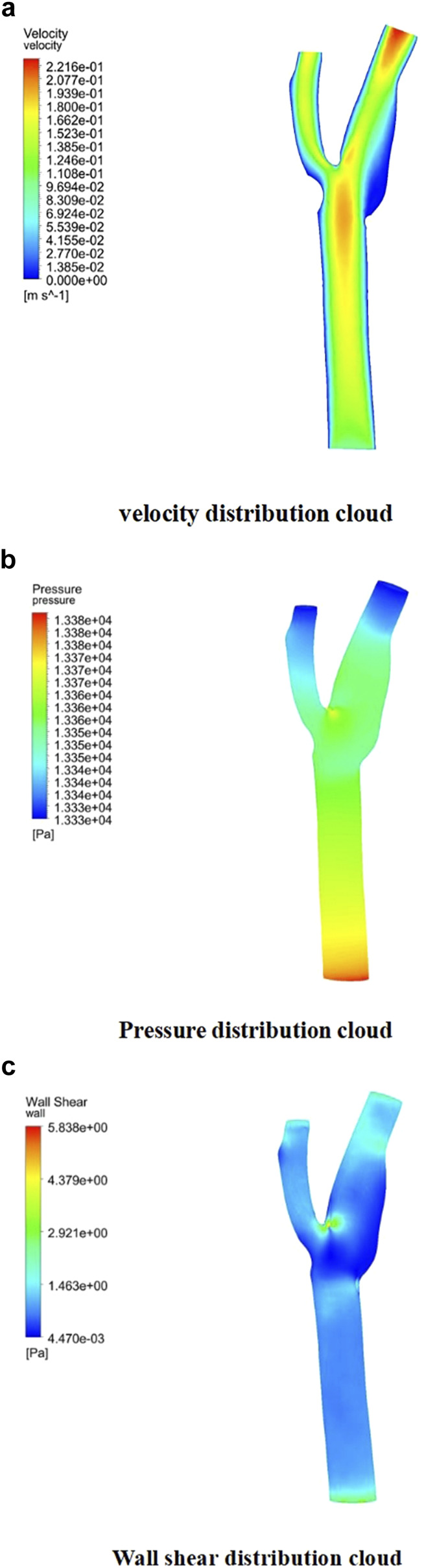
Plaque velocity, pressure, and shear force results in three locations. **(a)** Velocity distribution cloud. **(b)** Pressure distribution cloud. **(c)** Wall shear stress distribution cloud.

**FIGURE 4:**
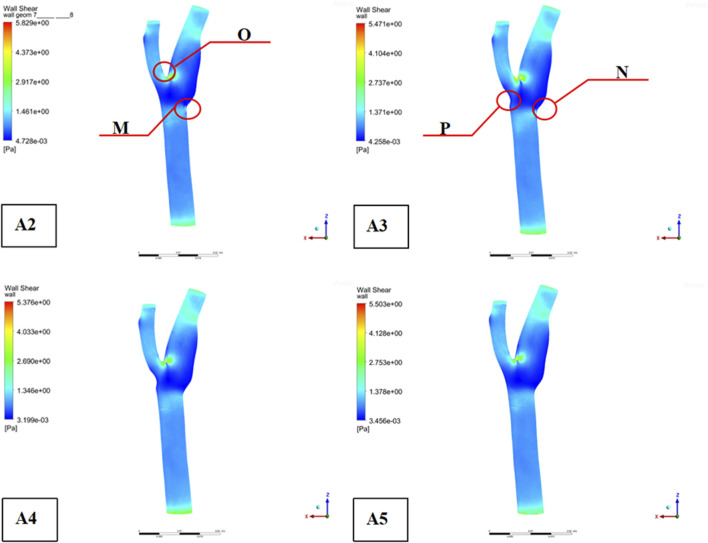
Distribution of shear forces on the wall ((“O” is located at the bifurcation ridge, “M” is the outer side of the common carotid artery (CCA), “N” is the outer side of the internal carotid artery (ICA), and “P” is the outer side of the external carotid artery (ECA)).

**FIGURE 5:**
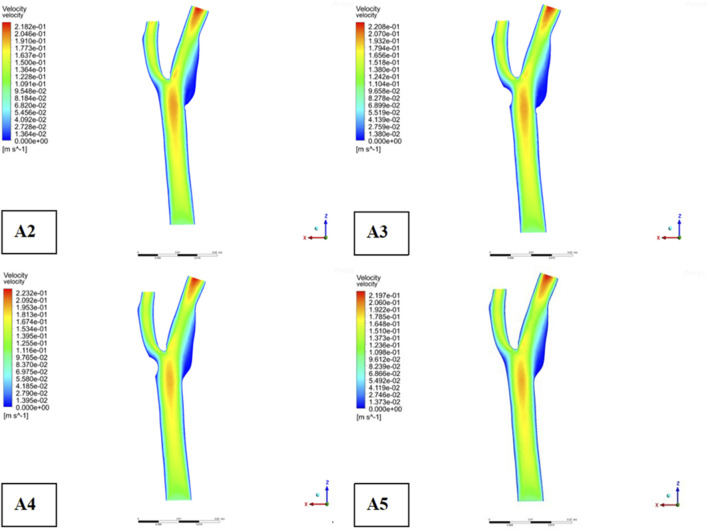
Middle section velocity distribution.

**FIGURE 6:**
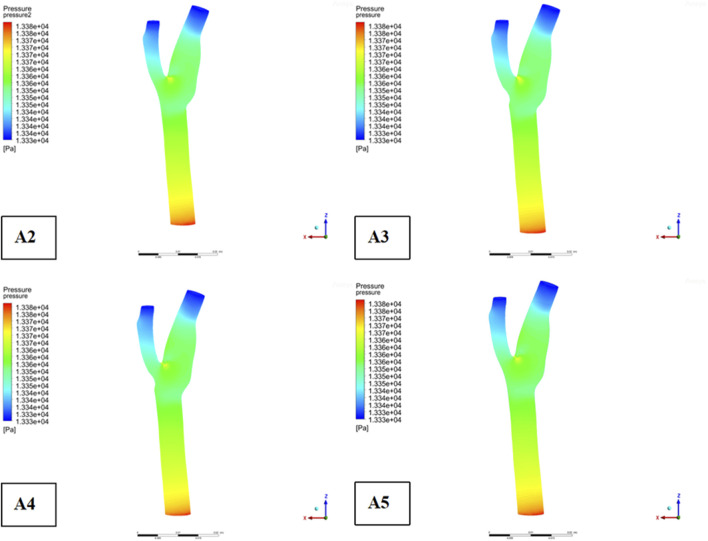
Wall pressure distribution.

Descriptive statistics were used to summarize key hemodynamic parameters (velocity, wall shear stress [WSS], and pressure) across different plaque configurations (Models A2-A5). Mean values and standard deviations were calculated for regions of interest, including upstream, downstream, and specific locations like the bifurcation ridge (M), shoulder region (N), and outer the carotid bifurcation(P). numerical simulations of hemodynamic parameters were conducted to investigate the impact of plaque formation at different locations within. the carotid bifurcation. Plaques were modeled at three key locations: the bifurcation ridge and the lateral fossa of the carotid bifurcation, where flow barriers were established to simulate plaque formation. The simulation results for various plaque locations are presented in Figure 3, while Figures 4–6 display the distribution maps of wall shear stress (WSS), velocity, and pressure at 1 s for models A2 to A5, respectively.

Across all four models, blood flow in the Common Carotid artery remained relatively stable, with lower velocities before encountering the plaque. However, upon reaching the plaque, significant changes were identify. Velocity increased, pressure dropped, and wall shear stress rose as blood passed through the plaque region. The maximum WSS occurred upstream of the plaque, designated as location “M” in the figures. Location “N,” representing the shoulder region just upstream of the plaque, also exhibited elevated WSS. These findings suggest that regions upstream of the plaque are susceptible to ulceration, rupture, and other types of mechanical damage, further promoting plaque progression.

On the outer side of the carotid bifurcation, at location “P,” a region characterized by low velocity and low shear stress was identify. Prior research indicates that WSS values below 0.5 Pa can facilitate the formation of atherosclerotic plaques, thus increasing the risk of plaque development at this site (Zhou M et al., 2023). Among the five plaque configurations analyzed, location “N,” corresponding to the upstream shoulder region, consistently exhibited higher WSS. This suggests a higher likelihood of ulceration and plaque rupture in this area, which could accelerate the progression of atherosclerosis.

As depicted in Figure 5, the location of plaques within the carotid bifurcation has a profound impact on the local hemodynamic environment. In Model A4, the presence of a plaque on the outer side of External carotid artery (ECA) significantly reduces downstream flow velocity, which in turn accelerates further plaque development. A concomitant increase in local pressure is identify in this region due to the reduced velocity. Additionally, Figure 5 illustrates that while blood flow is faster in the Common Carotid artery, it decelerates markedly at the bifurcation ridge, causing flow diversion into the branch vessels. The larger bifurcation angle of Internal carotid artery (ICA) leads to a low-velocity region on its outer wall.

Figure 6 highlights that this low-velocity region on the outer side of ICA corresponds to a high-pressure zone. Figure 4 further indicates that this region is also associated with low wall shear stress (WSS). Consequently, the outer region of ICA is characterized by a combination of low flow velocity, high pressure, and low WSS, making it a site highly prone to endothelial damage and atherosclerotic plaque formation.

**FIGURE 7:**
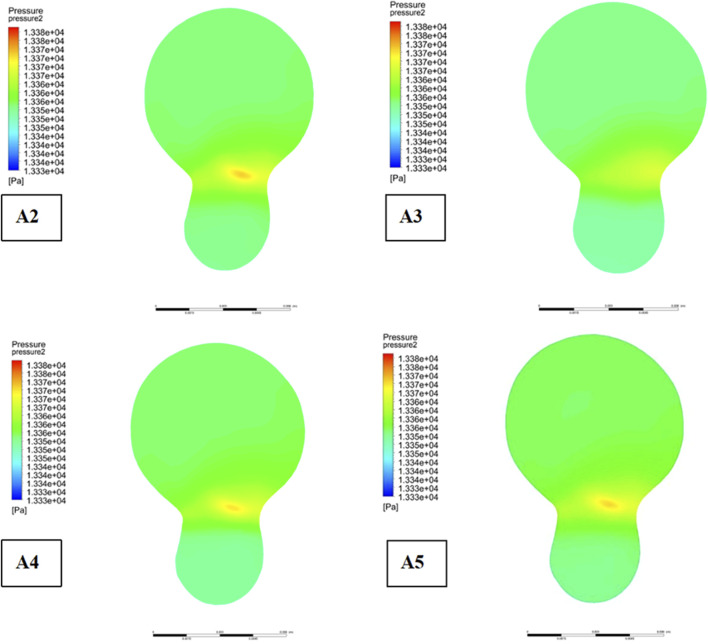
Pressure distribution at bifurcated ridges.

**FIGURE 8:**
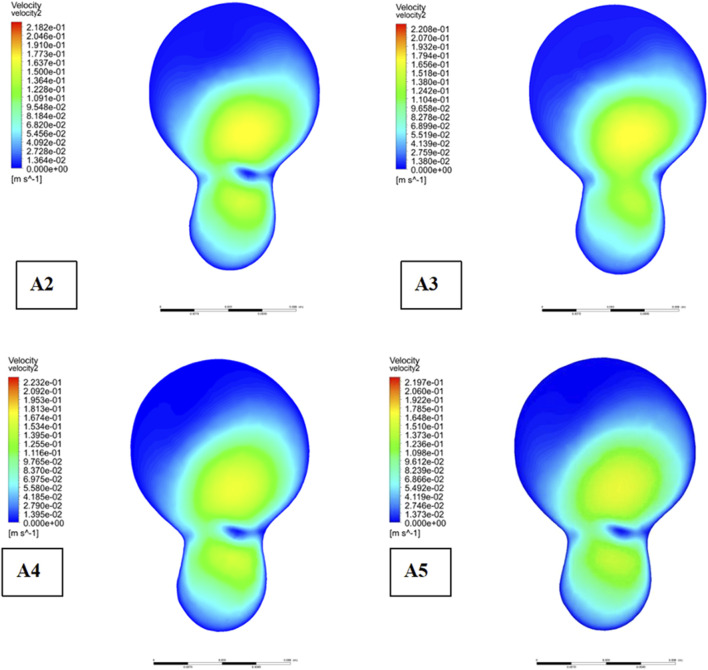
Flow velocity distribution at bifurcated ridges.

Figure 7 illustrates the comparative impact of different plaque types on the development of plaques at the bifurcation site. Meanwhile, Figure 8 provides an analysis of flow velocity and pressure distribution at the bifurcation ridge in models A2 through A5. The velocity vectors of the blood flow diverge at the bifurcation, moving towards the left and right branches, highlighting a shift in flow direction due to the interaction between the pressure exerted on the vessel wall and the wall shear stress (WSS). As the blood approaches the bifurcation ridge, the change in flow direction causes the ridge wall to bear the brunt of the flow impact. Consequently, a reduction in flow velocity and an increase in blood pressure occur at this point, leading to the formation of a high-pressure zone at the bifurcation.

A clear pattern can be seen by analysing Figure 8: in the presence of plaques, the velocity in the low-speed zone near the bifurcation ridge decreases significantly, while the maximum velocity in the mainstream zone increases, thus amplifying the velocity gradient across the entire cross-sectional area. This also results in greater pressure when plaques are present on both sides of the bifurcation ridge (as seen in Model A1, Figure 7), in contrast to the absence of plaques. These results suggest that the presence of plaques at the bifurcation increases pressure at the ridge while simultaneously reducing the flow velocity in the low-speed zone.

Moreover, the shear stress distribution across the cross-sectional area increases in the presence of plaques, indicating an enhanced risk of vessel wall damage. Notably, shear stress is more pronounced when plaques are located on one side of the bifurcation compared to plaques on both sides or no plaques. This suggests that when plaques are present on only one side of the bifurcation in CCA, the likelihood of plaque ulceration, rupture, and detachment is higher. Additionally, shear stress and pressure at the bifurcation ridge of CCA increase in the presence of plaques, supporting the notion that plaque presence within CCA further promotes plaque formation and progression at the bifurcation site.

### 3.2 Impact of different bifurcation angles

**FIGURE 9:**
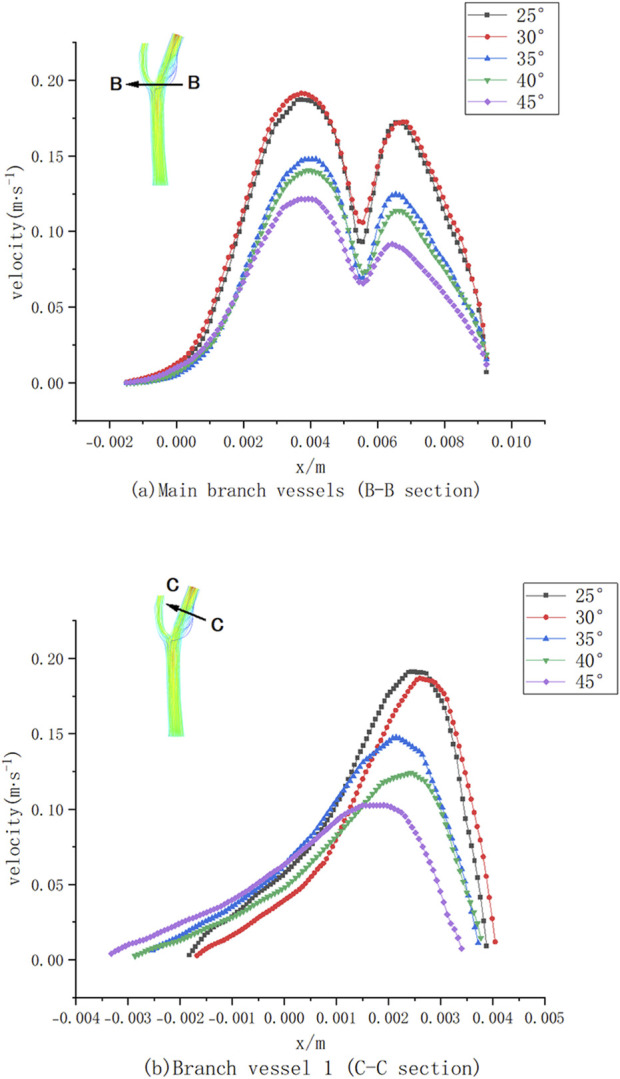
Cross-sectional velocity distribution curves of different bifurcation angle models.

Numerical simulations of five bifurcated carotid arteries with different bifurcation angles were performed to investigate the effects of these angles on haemodynamics and plaque formation. Figure 9 shows the velocity distribution curves of CCA (part B-B) and ICA (part C-C) at different bifurcation angles.

As illustrated in Figure 9A, the maximum flow velocity in ICA decreased by approximately 80% compared to CCA due to the increased effective flow area at the bifurcation. As the bifurcation angle increased, the maximum velocity at section B-B gradually decreased, corresponding to the expanded flow area at the bifurcation site. The velocity distribution curve exhibits two peaks, with a noticeable decline in maximum velocity at larger bifurcation angles. At a 45° bifurcation angle, the velocity in the branch vessels markedly decreased compared to that at a 25° angle, leading to a reduction in wall shear stress (WSS). However, larger bifurcation angles also resulted in the formation of low-velocity and low-pressure regions on the outer side of CCA, which are associated with an increased risk of atherosclerotic plaque formation.

Similarly, in Figure 9B, the maximum velocity in section C-C of ICA decreased with increasing bifurcation angles. At lower flow rates, the effects of blood viscosity became more pronounced, Higher blood viscosity increases the resistance to blood flow, leading to a slowdown in flow velocity, particularly in arterial branches or curved regions, leading to a substantial reduction in blood flow velocity on the inner side of ICA (0.004 m < x < 0.005 m) (Figure 9B) and the presence of a low-velocity zone on the outer side (−0.002 m < x < 0.002 m). With increasing bifurcation angles, the velocity within this low-velocity zone gradually increased, reflecting a complex interplay between bifurcation geometry and blood flow dynamics.

**FIGURE 10:**
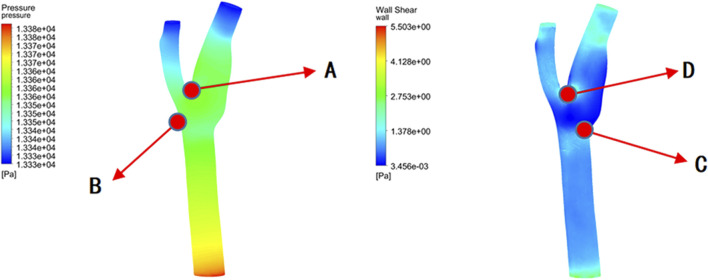
Shear stress and pressure extremes at different bifurcation angles.

Figure 10 reveals that the highest pressure values were concentrated at the bifurcation ridge (position A), while the maximum WSS values occurred on both sides of the bifurcation ridge (position D). Conversely, the lowest pressure and WSS values were identify on the outer side of the branch vessels (positions B and C, respectively).

**FIGURE 11:**
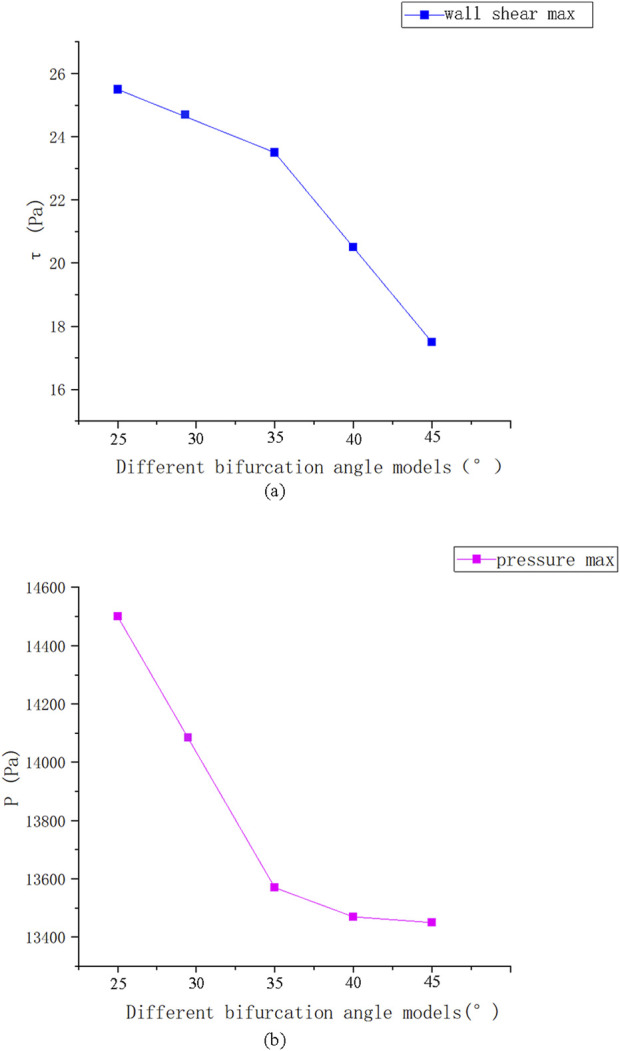
Extreme distribution of shear stress and pressure.

Figure 11 presents the instantaneous values of these hemodynamic parameters across different bifurcation angles. As the bifurcation angle increased, wall shear stress on both sides of the bifurcation ridge decreased, suggesting a reduced risk of mechanical damage. In contrast, at the point of minimum shear stress (position C) (Figure 10), the shear stress on the outer wall of ICA increased, implying a lower risk of damage in that specific region.

### 3.3 Distribution of cell residence time in carotid artery

Arterial regions characterized by vortex zones and low shear stress are particularly prone to thrombosis formation. In this study, we employed a discrete phase model to simulate the residence time distribution of blood cells within the carotid artery. The results suggest that blood cells tend to accumulate in regions of low shear stress, where endothelial cells are vulnerable to damage due to turbulent flow and reduced shear forces.

**FIGURE 12:**
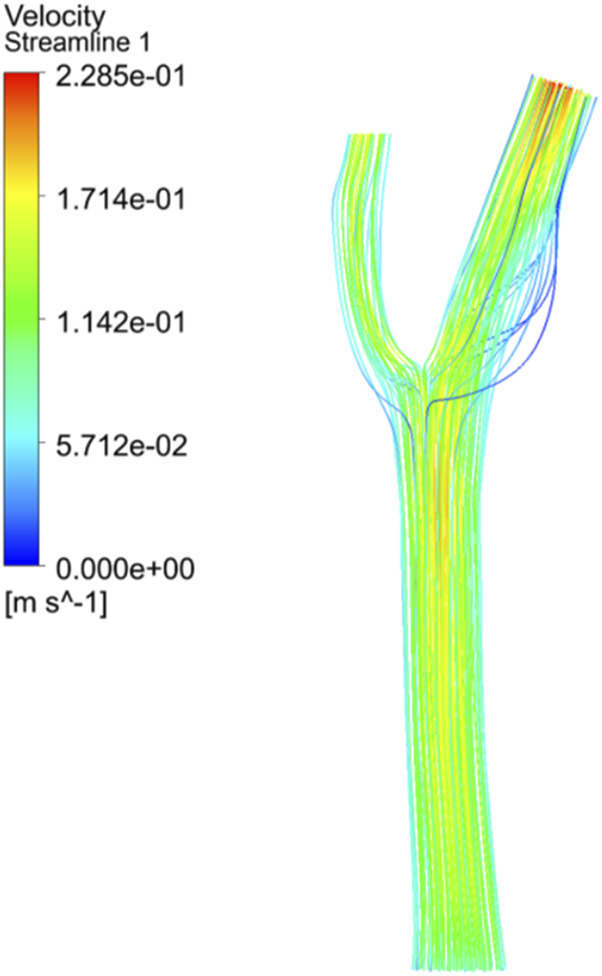
Movement trajectory of cells.

Our simulation results show a very different pattern of blood cell distribution between the two vessels. As shown in Figure 12, more blood cells enter ICA than ECA, especially at higher entrance velocities. Cells with longer residence times tended to attach to the vessel wall, whereas platelets flowed predominantly along the inner wall of the vessel. Prolonged contact between platelets, lipid components, and damaged endothelial cells favours deposition and promotes thrombosis.

Furthermore, we employed fluid-structure interaction (FSI) simulations combined with detailed computational fluid dynamics (CFD) models to investigate the interaction between pulsatile blood flow and the carotid artery wall. The primary goal was to elucidate the impact of fluid dynamic forces on arterial wall deformation and to explore the relationship between the distribution of blood flow patterns and the risk of plaque formation and rupture.

The results indicated that the deformation of the carotid artery wall is closely related to regions of high shear stress, particularly at bifurcation sites where flow patterns are more complex and exhibit clear turbulent characteristics. In these regions, the flow generates substantial mechanical stress on the artery wall, which may lead to endothelial injury and promote plaque formation. Furthermore, the coupling of fluid dynamics and structural mechanics highlighted the dynamic impact of blood flow characteristics, such as variations in inlet velocity, on the distribution of wall stress.

To validate the robustness of the model, we performed a parameter variation test for inlet flow velocities ranging from 0.3 to 0.7 m/s. These variations ensured that the model could adapt to different hemodynamic conditions and provided insights into how fluctuations in flow rate affect wall shear stress and the local mechanical environment of the artery. The analysis revealed that regions with low wall shear stress (<1 Pa) and vortex-like flow structures were identified as key areas for thrombosis formation, as these regions tend to promote the retention of blood cells, thereby increasing the risk of thrombus formation.

## 4 Discussion

This study investigates the complex hemodynamic mechanisms behind atherosclerotic plaque formation at vascular bifurcations. Consistent with previous research, our results show that plaque formation is primarily influenced by local hemodynamic stresses, particularly wall shear stress (WSS), and vessel geometry. Our simulations reveal that areas upstream of plaques with elevated WSS are more prone to mechanical injuries such as ulceration and rupture (Moerman et al., 2022).

We identified regions with low WSS, such as the outer wall of the external carotid artery (ECA) at location ‘P’, where WSS values below 0.5 Pa are more susceptible to plaque formation (Zhou et al., 2023). This aligns with the hypothesis that low WSS contributes to an atherogenic environment, where reduced mechanical forces on endothelial cells trigger maladaptive biological responses, including lipid retention, inflammation, and immune cell recruitment, all of which promote plaque development (Warboys and Weinberg, 2021).

The “N” location was identified as a high-risk site due to elevated WSS and its proximity to plaque formation, confirming the vulnerability of plaque shoulders to biomechanical stresses. Our findings suggest that the combination of high WSS and complex flow dynamics at bifurcations creates an environment that exacerbates plaque instability (Nooraeen et al., 2024). This is further supported by the presence of turbulence and oscillatory shear stress (OSI), which are known to induce endothelial dysfunction and increase plaque vulnerability (Thondapu et al., 2021).

The association between high WSS at “N” and increased risk of plaque rupture underscores the critical role of hemodynamic stress in plaque destabilization. High WSS and turbulence cause local structural weaknesses in plaques, making them more prone to rupture, which can result in serious clinical events such as thrombosis and embolism (Urschel et al., 2021).

This study also emphasizes the need for further exploration of complex blood flow patterns at bifurcations in relation to high-risk plaque features. Future studies should incorporate patient-specific geometries and consider the pulsatile nature of blood flow to more accurately model *in vivo* conditions. Investigating the relationship between WSS and plaque composition (e.g., lipid core and fibrous cap thickness) will provide deeper insights into plaque vulnerability and help develop targeted therapeutic strategies to reduce the risk of rupture and cardiovascular events.

The complex interplay between plaque location and local hemodynamic factors at bifurcated vessels is crucial (Han et al., 2024). We observed that plaque formation concentrated distally, particularly in the outer wall of the ECA in model A4, where reduced local flow velocities led to increased pressures and promoted plaque progression. This supports earlier findings that low flow velocity and high pressure downstream of plaques contribute to plaque growth and arterial stenosis (Bantwal et al., 2021).

Bifurcation ridges are high-risk areas for plaque formation and rupture (Kokkosis et al., 2021). The pattern of low-flow and recirculating flow near the bifurcation crest aligns with studies correlating low-flow with lipid deposition and endothelial dysfunction (Michel, 2020). This hemodynamic environment fosters atherosclerosis development. Moreover, the increased WSS and pressure at location ‘O’ further emphasize the role of biomechanical forces in plaque vulnerability, particularly at the plaque shoulder, where elevated WSS predisposes to intimal injury and rupture (Michel, 2020).

The identification of regions with low WSS, high pressure, and low flow velocity in the outer wall of the internal carotid artery (ICA) confirms that these conditions favor atherosclerosis formation. Low WSS promotes lipid retention, inflammation, and endothelial dysfunction, key factors in plaque formation (Zanoli et al., 2020). High pressure induces mechanical stress on the vessel wall, accelerating endothelial damage, plaque development, and increasing the risk of rupture (Nguyen et al., 2021).

The study highlights the importance of blood flow perturbations at bifurcation ridges, where complex low flow velocities, high pressures, and fluctuating WSS interact to create a biomechanical environment conducive to plaque formation, progression, and rupture. These ridges are particularly prone to low-velocity circulatory flow, which fosters the deposition of atherogenic substances like lipids and inflammatory cells. At the same time, elevated WSS and pressure cause mechanical damage to the intima, leading to platelet aggregation and inflammatory mediator accumulation (Franck, 2021).

Based on the properties of blood as a Newtonian fluid, characterized by homogeneity, viscosity, and incompressibility, this study classifies shear stress patterns in fluid dynamics, using the Navier-Stokes and continuity equations (Ren et al., 2025c). Specifically, shear stress patterns are categorized into two distinct states: laminar shear stress and oscillatory shear stress. Laminar shear stress is characterized by a time-independent directional blood flow vector distribution, whereas oscillatory shear stress is driven by the periodic pressure gradient reversal during the cardiac cycle, resulting in a time-varying amplitude vector field at the vessel wall. These dynamic characteristics reflect the complex flow patterns within the carotid system and their interaction with the vessel wall.

Oscillatory and turbulent shear stress patterns are pivotal in atherosclerosis pathogenesis, especially at bifurcations (Urschel et al., 2021). Oscillatory shear stress refers to time-varying hemodynamic forces with alternating directions, commonly observed in regions with flow separation and recirculation, while turbulent shear stress arises from blood flow fluctuations due to turbulence. These hemodynamic disturbances increase endothelial permeability, promote LDL accumulation, and trigger pro-atherosclerotic mechanisms. Oscillatory shear stress impairs endothelial function and induces inflammation, while turbulent shear stress exacerbates LDL retention and oxidation (Huang et al., 2022). Elevated WSS in regions of atherosclerotic plaques is linked to mechanical instability of the fibrous cap, increasing the risk of plaque rupture and thrombogenesis, underscoring the critical role of shear stress dynamics in atherosclerotic progression.

The bifurcation ridge is a key site of vulnerability to haemodynamic disturbances, especially in the presence of plaque. The domains where we identified reduced flow velocities and elevated pressures (Figures 7, 8) are the regions of prone to atherosclerosis. In contrast, low flow velocities and elevated wall shear stress (WSS) at bifurcation points enhance endothelial permeability and promote lipid infiltration and plaque formation (Silva et al., 2020). This study provides an important basis for understanding the haemodynamic changes and plaque progression at vascular bifurcations, especially bifurcation ridges.

In models with dense plaques (e.g., model A1), the localized increase in pressure at bifurcation ridges accelerates plaque development and instability, further reinforcing the importance of local mechanical stress in plaque progression. Models with unilateral plaques exhibit more localized mechanical stress compared to bilateral plaque models, consistent with evidence that asymmetry in plaque distribution increases the risk of rupture and ulceration (Hossain et al., 2024). These findings emphasize the impact of stress and plaque asymmetry on plaque vulnerability at bifurcations.

This study explores the complex hemodynamic interactions at bifurcation sites, which are key areas for atherosclerosis progression. Plaque formation at bifurcation crests creates a microenvironment characterized by reduced flow velocities and elevated pressure, promoting plaque development. Computational fluid dynamics (CFD) analyses suggest that these local flow alterations increase the likelihood of downstream plaque formation (Genuardi et al., 2021). Elevated wall shear stress (WSS) and pressure at these sites underscore the role of hemodynamics in both plaque progression and rupture risk (Naiya et al., 2024).

The bifurcation angle plays a critical role in regulating local hemodynamics, influencing plaque formation (iang et al., 2020). We found that larger bifurcation angles lead to lower flow velocities, which aligns with previous findings by Dong et al. (2015). This reduction in flow velocity creates extensive regions of low WSS, a known risk factor for lipid deposition and endothelial dysfunction. As the flow area at the bifurcation increases, branch vessel flow velocities decrease significantly, supporting CFD studies that demonstrate the significant impact of vessel geometry on local hemodynamics and atherosclerosis.

Our study confirms that larger bifurcation angles create conditions conducive to plaque formation, particularly in regions with disturbed flow and low shear stress, which promote lipid infiltration and atherogenesis. This observation is consistent with Chiu and Chien (2011), who found that low flow velocities and low shear stress contribute to plaque development. Reducing the bifurcation angle may help improve the local hemodynamic environment and mitigate atherosclerosis risk.

Furthermore, the study highlights the importance of extreme hemodynamic conditions, such as elevated WSS and pressure, in atherosclerosis pathogenesis. High-pressure zones at bifurcation ridges, combined with elevated WSS, emphasize the role of mechanical stress in plaque formation and potential instability, consistent with Eshtehardi et al. (2017), Dong et al. (2015). In contrast, low-pressure areas on the branch vessel’s lateral aspects are prone to plaque accumulation due to disturbed hemodynamics.

The vortex zone, marked by turbulent and recirculating flow, contributes to endothelial dysfunction, platelet adhesion, and thrombosis. Our findings show greater blood cell accumulation in the external carotid artery (ECA) than in the internal carotid artery (ICA), suggesting asymmetry in blood flow at arterial branches, which enhances thrombosis risk. Prolonged residence time of blood cells in regions of low shear stress further promotes platelet adhesion and thrombus formation (Owais et al., 2023; Alkarithi et al., 2021). This phenomenon, seen in areas of endothelial injury, creates a pre-thrombotic environment, heightening the risk of thrombus instability and rupture, which can lead to severe clinical events like ischemic stroke or internal bleeding (Kotlyarov, 2022; Bray et al., 2020; Miele et al., 2024).

Atherosclerosis progresses through distinct stages influenced by hemodynamic forces. Early-stage plaque formation is characterized by disturbed flow dynamics, particularly low time-averaged WSS (TAWSS) and high oscillatory shear index (OSI), which are prevalent at bifurcations (Peiffer et al., 2013). These conditions lead to endothelial dysfunction and lipid infiltration, promoting plaque formation. The CFD model identifies regions with low TAWSS (<0.5 Pa) and high OSI (>0.3), where sluggish and oscillatory flow accelerates atherogenesis. Variations in bifurcation angle (25°–45°) exacerbate flow separation, further increasing plaque risk.

Plaque rupture occurs in advanced atherosclerosis, when mechanical stresses exceed the fibrous cap’s structural integrity (Moerman and Korteland, 2022). This is associated with high WSS, particularly peak systolic WSS (>7 Pa), which destabilizes the plaque and leads to rupture. The CFD model shows that high WSS and OSI, combined with low TAWSS, indicate regions vulnerable to plaque destabilization.

Risk stratification using CFD parameters identifies high-risk plaques characterized by low TAWSS, high OSI, and increased endothelial cell activation potential (ECAP >1.5), which are more prone to rupture. Conversely, plaques with high TAWSS and low OSI are considered low-risk (Mutlu and Salman, 2023).

The integration of fluid-structure interaction (FSI) simulations with CFD provides valuable insights into the mechanical and biological processes at play in carotid artery remodeling (Syed et al., 2023). Our findings confirm that arterial wall deformation is non-uniform, with significant strain at bifurcation zones where turbulent flow and high OSI occur. These regions show a correlation between peak systolic WSS (>7 Pa) and arterial dilation, suggesting that cyclic mechanical loading contributes to endothelial dysfunction and extracellular matrix remodeling. The persistence of vortical flow patterns and flow reversal enhances monocyte adhesion, lipid infiltration, and platelet activation, core processes in atherogenesis (Linton et al., 2019). These CFD findings are consistent with histopathological observations of plaque rupture at flow stagnation zones in human carotid specimens (Savastano et al., 2022).

Our parametric analysis reveals a non-linear relationship between inlet velocity modulation (0.3–0.7 m/s) and circumferential stress gradients, underscoring the dynamic interplay between pulsatility and arterial wall fatigue. This feedback loop may exacerbate vascular remodeling in hypertensive states.

Clinically, the spatial mapping of critical WSS thresholds and vorticity metrics derived from this framework holds potential for stratifying rupture risk in carotid atherosclerotic plaques. By correlating computational predictions with intraoperative observations of plaque morphology and vulnerable features (e.g., thin-cap fibroatheroma, intraplaque hemorrhage), such techniques may refine preoperative planning for carotid endarterectomy or stenting. Moreover, the demonstrated sensitivity of wall stress distribution to inlet velocity variability underscores the importance of rigorous blood pressure management in mitigating hemodynamically driven vascular injury.

While this model effectively captures key hemodynamic-biomechanical interactions, some limitations remain. The assumption of isotropic arterial wall properties may underestimate stress heterogeneity in diseased segments, and the exclusion of transient inflammatory responses and endothelial glycocalyx dynamics limits the model’s ability to fully capture the biological response to shear-induced injury. Future studies incorporating patient-specific geometries, multiphase flow modeling, and *in vivo* validation via 4D flow MRI could further refine these findings.

## 5 Conclusion

This computational study investigates the hemodynamic and biomechanical factors influencing plaque vulnerability at carotid bifurcations. Utilizing a CFD-FSI framework based on high-resolution 3D imaging, we identify significant correlations between altered wall shear stress (WSS), oscillatory flow patterns, and localized arterial remodeling. Our key findings reveal that bifurcation geometry—particularly variations in angle (ranging from 25° to 45°)—modulates hemodynamic disturbances, enhancing regions with low time-averaged WSS (TAWSS) and high oscillatory shear index (OSI), which are conducive to plaque formation, while also exacerbating high WSS zones, which are susceptible to fibrous cap rupture. The integration of hemodynamic parameters, including TAWSS <0.5 Pa, OSI >0.3, and ECAP >1.5, offers a novel risk stratification method for assessing plaque vulnerability. In conclusion, this multiscale modeling approach sheds light on the mechanopathological continuum connecting disturbed flow patterns with structural vessel deterioration, providing a quantitative foundation for developing targeted therapeutic strategies aimed at modulating biomechanical risk factors in carotid atherosclerosis.

### 5.1 Strengths and limitations

The primary strength lies in the multiscale modeling of pulsatile flow-biomechanical coupling, validated against clinical observations of plaque localization and rupture mechanics. However, the idealized geometric assumptions and static boundary conditions oversimplify anatomical variability and dynamic vascular compliance. The exclusion of biological factors (endothelial reactivity, inflammatory cascades) and systemic disease influences (hypertension, diabetes) restricts physiological relevance.

### 5.2 Future Directions

Advancements require patient-specific 3D reconstructions incorporating dynamic cardiac cycles, anisotropic vessel properties, and endothelial pathobiology. Validating computational predictions with 4D flow MRI and histopathological correlations will bridge mechanistic insights to clinical applications. Addressing these limitations through fluid-structure-immune interaction models could refine risk prediction and therapeutic strategies for carotid atherosclerosis.

## Data Availability

The original contributions presented in the study are included in the article/supplementary material, further inquiries can be directed to the corresponding author.
